# Special Issue “Adipokines, Myokines, and Physical Exercise in Health and Disease 2.0”

**DOI:** 10.3390/ijms25020940

**Published:** 2024-01-11

**Authors:** Jan Bilski, Tomasz Brzozowski

**Affiliations:** 1 Department of Biomechanics and Kinesiology, Institute of Physiotherapy, Faculty of Health Sciences, Jagiellonian University Medical College, 31-008 Cracow, Poland; 2 Department of Physiology, Faculty of Medicine, Jagiellonian University Medical College, 31-531 Cracow, Poland

We are pleased to present our Editorial to this Special Issue on “Adipokines, Myokines, and Physical Exercise in Health and Disease 2.0” published by IJMS. The intricate interplay between adipose tissue, skeletal muscle, and exercise has become a fascinating research area with profound implications for human physiology and the treatment of various diseases [1,2]. Participation in physical activity and regular exercise play an essential role in the prevention and management of chronic diseases such as cardiovascular disease, obesity, type 2 diabetes, cognitive impairment, and various forms of cancer. These activities improve the immune system, extend the health system, promote longevity, and foster resilience [3].

By examining the roles of adipokines and myokines, we recognised the evolving importance of these molecular messengers. Skeletal muscles, which make up approximately 40% of the body mass, have more than a simple mechanical power. As Legård and Pedersen [4] suggested, it is one of the largest endocrine organs alongside white adipose tissue [5,6]. Our understanding of the muscle as an endocrine organ has expanded with the discovery of myokines that exert paracrine and endocrine effects, including proteins, miRNA, and exosomes [7]. Skeletal muscles secrete numerous myokines, including myostatin, IL-4, IL-6, IL-7, IL-15, myonectin, follistatin-like 1, leukemia inhibitory factor, and irisin [1,8]. These myokines act locally in the muscle and regulate physiological processes in other tissues. The release of myokines from contracting muscle contributes to the health-promoting effects of physical activity, protecting against low-grade inflammatory diseases, such as type 2 diabetes, insulin resistance, and metabolic syndrome [8].

Obesity, characterised by an excessive accumulation of adipose tissue, is an increasing societal concern with detrimental health implications. Adipose tissue, a complex metabolic organ comprising brown adipose tissue (BAT) and white adipose tissue (WAT), plays a crucial role in energy homeostasis [9]. Adipose tissue is more than just a fat store and a place of lipid metabolism; it functions as a dynamic endocrine organ. It produces a range of bioactive substances, including adipokines, which are key in controlling the overall metabolism [10,11,12].

Dysregulation associated with obesity precipitates a state of chronic low-grade inflammation and alters adipokine production, increasing the risk of metabolic and cardiovascular diseases [13,14], as well as certain malignancies [15].

The increasing prevalence of lifestyle-related diseases underscores the potential significance of crosstalk between adipose tissue and skeletal muscle, a dialogue that may be pivotal for understanding and combating these health conditions.

In this Special Issue, we delve deeper into the intricate world of adipokines and myokines and their interactions with physical exercise. Our goal was to bridge the gap between the scientific understanding of these molecular messengers and their practical implications for health and disease management. The articles in this issue provide a comprehensive overview of recent advances and developments, highlighting the promising use of adipokines and myokines as therapeutic targets. The articles presented in this Special Issue shed light on the molecular intricacies of exercise from various perspectives.

## An Overview of Published Articles

The role of the mitochondrially derived MOTS-c peptide in the regulation of nuclear responses during exercise-induced stress was investigated by Domin et al. A positive correlation was found between MOTS-c levels and lower body muscle strength, suggesting that higher MOTS-c levels may enhance explosive strength. MOTS-c was also associated with total muscle mass and BMI, but not with maximal oxygen uptake (VO_2_ max), indicating that it may not directly affect muscle endurance. Previous studies revealed that MOTS-c can act as a mitochondria-derived peptide (MDP) and a potential myokine, which is released in response to exercise and is involved in energy metabolism [16]. MOTS-c may facilitate communication between muscles and other organs, contributing to energy balance during exercise; however, there is a need for further research into the role of MOTS-c in exercise physiology.

Cathelicidin antimicrobial peptide (CAMP), a potent antimicrobial peptide produced by adipocytes, is a cornerstone of the innate immune defence system within subcutaneous AT, specifically targeting Gram-positive bacterial pathogens [17]. The study by Höpfinger et al. adds complexity by examining the regulation of cathelicidin antimicrobial peptide (CAMP) and its functions as an adipokine. A study of 86 metabolically healthy participants examined postprandial variations, providing insight into the role of CAMPs in defence against infection and innate immunity in adipose tissue. Circulating CAMP levels increased shortly after oral glucose ingestion. Moreover, the differences in glucose-related systemic CAMP regulation were observed between men and females, as well as between normal-weight and overweight individuals, which indicated a link between glucose metabolism and the immune-metabolic factor CAMP, thus helping to understand metabolic dysregulation and immune modulation in adipose tissue. However, the question concerning the specific effects on CAMP regulation remains, which warrants further investigation.

The role of apelin as a cardiokine/myokine, a signaling molecule, in the regulation of cardiac and skeletal muscle homeostasis during peak athletic performance has not been fully explained [18]. Ligetvári et al. explored the role of apelin as a cardiokine/myokine, a signaling molecule, in regulating cardiac and skeletal muscle homeostasis during peak athletic performance. The effect of maximal cardiorespiratory exercise testing on the plasma concentrations of apelin-13 and apelin-36 in professional soccer players was investigated. The results showed that apelin-13 levels increased transiently after exercise, with significant inter-individual variability. Baseline apelin-13 levels were inversely correlated with exercise-induced changes and positively correlated with indices of physical performance. Apelin-13 may be a determinant of peak athletic performance and is considered an index of physical effort, offering novel insights into the role of apelin in exercise physiology. This implies the need for further research in the field of sports medicine.

Sarcopenia is known as a disease characterised by a decrease in muscle mass, functionality, and strength loss associated with aging; however, its complex relationship with obesity and metabolic consequences in the context of exercise in mitigating these effects should be examined [19,20]. Recent developments in this field, including the functional assessment of organokines in sarcopenia, indicated an important role of these molecules in the pathophysiology and metabolic sequelae of sarcopenia [20]. Minniti et al. comprehensively studied sarcopenia, a disease characterised by muscle mass, functionality, and strength loss associated with aging. The authors highlighted the complex relationship between sarcopenia, obesity, and metabolic consequences, emphasising the role of exercise in mitigating these effects. They discussed the function of organokines in sarcopenia and explored how these molecules contribute to the disease’s pathophysiology and metabolic sequelae. The authors concluded by emphasising the significance of understanding the physiology of organokines in unravelling the molecular aspects of sarcopenia and highlighting the role of physical exercise as a non-invasive intervention for sarcopenic obesity.

Nintou et al. undertook a unique approach by analysing the in vitro protocols used for the electrical pulse stimulation of cultured cells to mimic in vivo exercise. The research conducted a systematic review and meta-analysis of studies using EPS to mimic exercise in cell cultures, contributing to our understanding of the potential of in vitro exercise as a tool for discovering mechanistic effects. The in vitro protocols used for the electrical pulse stimulation (EPS) of cultured cells that mimic in vivo exercise are a unique approach to analyse the mechanistic effects of exercise-induced health benefits [21]. A total of 985 records were screened, resulting in 41 eligible studies in which an in vitro exercise showed considerable heterogeneity in terms of duration, type (acute or chronic), and intensity (aerobic, resistance, and endurance). Key biological parameters related to exercise, including AMPK, Akt, IL-6, PGC1a, GLUT4, and glucose uptake, were identified. For example, in vitro exercise using EPS follows the exercise motifs in humans, demonstrating a significant impact on key biological parameters related to exercise. Further research is recommended to achieve a consensus and improve the translation of these findings to human studies. Understanding the molecular responses in cell cultures can help unravel the effects of exercise, which could lead to improved applications in health and disease research. The beneficial effects of exercise may depend on its intensity [22], and Wojcik-Grzybek et al. documented that forced exercise using treadmill running exacerbated experimental colitis in mice fed a high-fat diet to induce obesity. Interestingly, the exogenous administration of intestinal alkaline phosphatase (IAP) attenuated experimental colitis in obese mice subjected to forced treadmill exercise. IAP administration resulted in a significant reduction in pro-inflammatory biomarkers, attenuation of oxidative stress markers, improved gene expression of intestinal barrier tight junction proteins, and modified intestinal microbiota. A particularly noteworthy observation was that IAP treatment in obese mice with colitis was manifested by a significant reduction in plasma leptin levels, suggesting that IAP may not only attenuate inflammation but also potentially contribute to weight control in overweight patients who want to lose weight but suffer from lower gastrointestinal diseases, such as IBD, which may worsen because of intense physical exercise. Exercise intensity plays a crucial role in attaining human health benefits; notably, very low-volume interval training, as opposed to high-intensity exercise training, improved non-alcoholic fatty liver disease and overall cardiometabolic health in patients with obesity and metabolic syndrome [18]. Thus, IAP may be an alternative to pharmacological agents as a therapeutic option for the treatment of IBD, especially in obese patients who would like to lose weight urgently. IAP treatment can also improve the quality of life of these patients by reducing the severity of colitis and mitigating the negative effects of excessive exercise. Further research is needed to fully elucidate the mechanisms underlying the beneficial effects of IAP and to confirm the IAP efficacy in ameliorating colitis in the clinical setting. Nevertheless, the investigation of IAP as a new therapeutic approach for the treatment of IBD deserves attention.

We sincerely appreciate the authors’ contributions and are confident that this Special Issue will spark further scientific interaction and researcher dialogue. Taken together, these collective advances improve our understanding of the intricate interactions between adipokines and myokines concerning health and disease. Elucidating the signaling mechanisms of these substances holds great potential for the development of novel biomarkers and targeted therapies for the treatment of common diseases, such as obesity, diabetes, and sarcopenia.

Future research should prioritize examining the impact of intervention on the gut microbiome and its role in regulating the myokine–adipokine profile. To successfully translate these findings, it is necessary to bridge the gap between animal models and human clinical settings. As highlighted in this issue, much more remains to be understood regarding adipokines, myokines, and their complex interactions affecting physiology and well-being. We hope that the articles published in this Special Issue will further illuminate this captivating area at the intersection of metabolism, immunology, microbiology, and exercise science.
